# Supplementary material for: Roles of the Crp/Fnr Family Regulator ArcR in the Hemolysis and Biofilm of Staphylococcus aureus
Source: Microorganisms. 2023 Jun 25;11(7):1656. doi: 10.3390/microorganisms11071656 (PMC10384999; doi:10.3390/microorganisms11071656)
Supplement: Supplementary file 1 [file microorganisms-11-01656-s001.zip › microorganisms-2448950-supplementary.pdf]

Supplementary Table S1 Primers used in this study.

Name	Sequence (5'-3')	Purpose
R*arcR*-UP-F-EcoRI	ATCGCAGTGCAGCGGAATTCTTGTGGTGCAATGTCACAGGGTATG	*arcR* deletion
R*arcR*-UP-R	TGTTTGCTTACTAACCAATCTGCTAATGCCTTTAGTTCATG	
R*arcR*-DN-F	CATGAACTAAAGGCATTAGCAGATTGGTTAGTAAGCAAACA	
R*arcR*-DN-R-HindIII	AAACTACCGCATTAAAGCTTAGCATCAGCAGCATTTACTACCG	
R*icaA*-UP-F-EcoRI	ATCGCAGTGCAGCGGAATTCAGTAGCGAATACACTTCATC	*icaA* deletion
R*icaA*-UP-R	TCCAGTATACTGTCGGATACGACAACGTATCTTCAATCGT	
R*icaA*-DN-F	ACGATTGAAGATACGTTGTCGTATCCGACAGTATACTGGA	
R*icaA*-DN-R-HindIII	AAACTACCGCATTAAAGCTTAAGCCATAAGGATAGGCTATAG	
pCN51-*arcR*-F	GGTCAATGTCTGAACCTGCAGAGTTGTCTGCTGACACTTTGC	Expression of *arcR* in *S. aureus*
pCN51-*arcR*-R	TCCTCTAGAGTCGACCTGCAGTACGTTAGACCTCATGTTCAAC	
pOS1-P*hla*F	CAAAGCCTTAAAGACGATCCGGGGAATTCCCGACGAAATTCCAAACATA	β-Galactosidase activity assay of *hla* promoter
pOS1-P*hla*R	TCACGACGTTGTAAAACGACGGGATCCGGCATTAGCGACAGGATT	
pOS1-P*hlb*F	CAAAGCCTTAAAGACGATCCGGGGAATTCTGCAACTTAATTATAGCCAG	β-Galactosidase activity assay of *hlb* promoter
pOS1-P*hlb*R	TCACGACGTTGTAAAACGACGGGATCCTGCTAATGCAAGTGTTGCAAC	
pOS1-P*sbi*F	CAAAGCCTTAAAGACGATCCGGGGAATTCTCTACTCTACCAAACTCTCTTCG	β-Galactosidase activity assay of *sbi* promoter
pOS1-P*sbi*R	TCACGACGTTGTAAAACGACGGGATCCAGCTAACGTAATTGTTGCTG	
pOS1-P*vraX*F	CAAAGCCTTAAAGACGATCCGGGGAATTCACTCATCTATATGCTCAATACCTC	β-Galactosidase activity assay of *vraX* promoter
pOS1-P*vraX*R	TCACGACGTTGTAAAACGACGGGATCCTTCATGGTGATACTGTCGAT	
pOS1-P*splA*F	CAAAGCCTTAAAGACGATCCGGGGAATTCACATGAATTGTTCACCATTCC	β-Galactosidase activity assay of *splA* promoter
pOS1-P*splA*R	TCACGACGTTGTAAAACGACGGGATCCAGCAGTTAAACCTTTAACC	
pET28a-*arcR*-F	TTGAAGGAGTTTAACTTATGCATCATCATCATCATCACACAGAAAACTTTATTTTGG	Expression of *arcR* in *E. coli*

pET28a-*arcR*-R	TCCTCTAGAGTCGACCTGCAGTACGTTAGAC CTCATGTTCAAC	
Q-16S rRNA-F	ACAAAGTGACAGGTGGTGCA	qRT-PCR
Q-16S rRNA-R	GTTTGTCACCGGCAGTCAAC	
Q-*hla*-F	ACAACACTATTGCTAGGTTCC	qRT-PCR
Q-*hla*-R	TCGTTCTAATAACTAGCAG	
Q-*hlb*-F	AGTTGCAACACTTGCATTAGC	qRT-PCR
Q-*hlb*-R	AAGACTATCGAAGCAAGCG	
Q-*icaA*-F	TACGTTGTCTAATGTTCTTGC	qRT-PCR
Q-*icaA*-R	AGTATCTGCATCCAAGCAC	
Q-*icaB*-F	TGCAGATGACGATTCACC	qRT-PCR
Q-*icaB*-R	TAGCATCATGTGATTTTAGCC	
Q-*icaC*-F	TCTTGTCACAGTTACTGACAACC	qRT-PCR
Q-*icaC*-R	TACCATTGACCTAATAGGAC	
Q-*icaD*-F	AGAGAAACAGCACTTATCGC	qRT-PCR
Q-*icaD*-R	AGCAACACGTATTGTATTG	
Q-*sbi*-F	ACAACTACGTAACAGATCAAC	qRT-PCR
Q-*sbi*-R	TGCAACACGTCGGTCTGGGTTC	
Q-*vraX*-F	ATCGACAGTATCACCATGAAGG	qRT-PCR
Q-*vraX*-R	CATATGATCTATATCGTCTTG	
Q-*splA*-F	TCAGCCTCATTCAATTGCC	qRT-PCR
Q-*splA*-R	ACTCGAATGATGTGCTGATACTC	
Q-*yycF*-F	ACCTGGTCGTGATGGTATGG	qRT-PCR
Q-*yycF*-R	CCAGTGTCTTGTGCTGGTTG	
Q-*yycG*-F	ACCAGACGGTCGAAATGTCC	qRT-PCR
Q-*yycG*-R	ACCAAGCATCTTGAGTGCC	
E-*icaA*-F	AGCTATATCATCAAGTGTTG	EMSA
E-*icaA*-R	ACCTACCTTTCGTTAGTTAG	
E-*hla*-F	TCAACTTTGACTAACCCTCG	EMSA
E-*hla*-R	AACCTAGCAATAGTGTTGTTG	
E-*hlb*-F	AGTTGTTGTAATCAATGAC	EMSA
E-*hlb*-R	TGCTAATGCAAGTGTTGCAAC	
E-*tetA*-F	AGCGCGTGTTGTTATGTCGC	EMSA
E-*tetA*-R	ACCTCCTTATTAGACAATGTG	

Supplementary Table S2 The quantitative hemolytic activity

Samples	OD543		
NCTC8325	0.786	0.963	0.910
Δ*arcR*	1.505	1.488	1.400
Δ*arcR*/*arcR*	0.700	0.665	0.788
Negative	0.087	0.035	0.071
Positive	1.750	1.663	1.575

Supplementary Table S3 Genes downregulated by ArcR. ($P < 0.05$ and fold change $log_2 <-2$)

Gene_id	Log2Fold Change	Gene_name	Gene_description
NCTC8325_02680	-7.12	*arcA*	Arginine deiminase
NCTC8325_00169	-5.54	*pflB*	formate acetyltransferase
NCTC8325_00183	-5.27	*hmp*	Flavohemoprotein (Hemoglobin-like protein)
NCTC8325_00170	-5.26	*pflA*	Pyruvate formate-lyase activating enzyme
NCTC8325_01308	-5.41	*ald1*	Alanine dehydrogenase
NCTC8325_01307	-5.29	*ilvA*_1	Threonine dehydratase%2C catabolic
NCTC8325_00181	-5.31	NCTC8325_00181	Putative cytosolic protein
NCTC8325_00182	-4.86	NCTC8325_00182	membrane protein
NCTC8325_01306	-4.89	*steT*	amino acid permease
NCTC8325_00202	-4.63	*scdA*	DnrN
NCTC8325_02679	-4.98	*arcB*	ornithine carbamoyltransferase
NCTC8325_02660	-4.52	NCTC8325_02660	Ribonucleotide reductase of class III
NCTC8325_02661	-4.13	*nrdD*	Ribonucleotide reductase of class III
NCTC8325_02526	-3.99	NCTC8325_02526	Uncharacterised protein
NCTC8325_00099	-3.77	*adhE*	acetaldehyde dehydrogenase
NCTC8325_02753	-4.76	NCTC8325_02753	Permease
NCTC8325_00949	-4.07	NCTC8325_00949	Cytochrome d ubiquinol oxidase subunit II
NCTC8325_00557	-3.35	*adh1*	Alcohol dehydrogenase
NCTC8325_01027	-3.56	*argF*	ornithine carbamoyltransferase
NCTC8325_01305	-3.18	*norB*_4	Multidrug resistance protein B
NCTC8325_01311	-3.38	NCTC8325_01311	pepSY-associated TM helix family protein
NCTC8325_00948	-3.21	*cydA*	Cytochrome d ubiquinol oxidase subunit I
NCTC8325_02594	-2.76	*clpL*	putative ATP-dependent protease ATP-binding subunit ClpL
NCTC8325_00180	-2.63	*gsiB*	ABC superfamily ATP binding cassette transporter
NCTC8325_02678	-2.64	*arcD*_2	arginine/ornithine antiporter PF13520:Amino acid permease
NCTC8325_00055	-2.60	NCTC8325_00055	myosin-crossreactive antigen

NCTC8325_02752	-2.60	NCTC8325_02752	membrane protein
NCTC8325_02751	-2.22	*immR*_2	Transcriptional regulator
NCTC8325_01028	-2.34	*arcC1*	carbamate kinase

Supplementary Table S4 Genes upregulated by ArcR. ($P < 0.05$ and fold change $log_2 > 2$)

gene_id	log2Fold Change	gene_name	gene_description
NCTC8325_01872	13.60	NCTC8325_01872	Phage head protein
NCTC8325_01873	13.06	NCTC8325_01873	phi 11 orf33 && PF14265
NCTC8325_01870	11.82	NCTC8325_01870	phi 11 orf36 && PF05135
NCTC8325_01869	11.54	NCTC8325_01869	phage-like protein
NCTC8325_01875	11.49	NCTC8325_01875	phage putative head morphogenesis protein
NCTC8325_01868	11.38	NCTC8325_01868	phi 11 orf37 && PF04883
NCTC8325_01871	11.18	NCTC8325_01871	phi 11 orf35 && -
NCTC8325_01874	8.63	NCTC8325_01874	Uncharacterised protein && -
NCTC8325_00457	6.77	NCTC8325_00457	16S ribosomal RNA
NCTC8325_00571	5.97	NCTC8325_00571	Protein of uncharacterised function (DUF2922)
NCTC8325_02348	4.5	NCTC8325_02348	Uncharacterised protein
NCTC8325_01867	4.43	NCTC8325_01867	phi 11 orf38
NCTC8325_01188	4.12	NCTC8325_01188	Uncharacterised protein
NCTC8325_01707	4	NCTC8325_01707	comK family protein
NCTC8325_01408	3.66	NCTC8325_01408	phage protein
NCTC8325_01982	3.46	*chp*	Chemotaxis-inhibiting protei
NCTC8325_02713	3.45	*icaB*	polysaccharide deacetylase
NCTC8325_01979	3.44	*hlb*_1	beta-hemolysin
NCTC8325_01411	3.34	NCTC8325_01411	DNA-binding protein
NCTC8325_02649	3.15	NCTC8325_02649	Uncharacterised protein
NCTC8325_01876	3.03	NCTC8325_01876	phage-like protein
NCTC8325_00172	2.96	*scn*_1	complement inhibitor SCIN family protein
NCTC8325_02711	2.87	*icaA*	Polysaccharide intercellular adhesin (PIA) biosynthesis N-glycosyltransferase IcaA
NCTC8325_02722	2.74	*hisD*	Histidinol dehydrogenase
NCTC8325_01415	2.66	NCTC8325_01415	putative phage transcriptional regulator
NCTC8325_01749	2.63	NCTC8325_01749	probable beta-lactamase
NCTC8325_00453	2.62	NCTC8325_00453	16S ribosomal RNA
NCTC8325_02724	2.55	NCTC8325_02724	ATP phosphoribosyltransferase%2C regulatory subunit
NCTC8325_00247	2.53	NCTC8325_00247	lipoprotein%2C putative

NCTC8325_01828	2.52	NCTC8325_01828	23S ribosomal RNA
NCTC8325_02208	2.51	NCTC8325_02208	16S ribosomal RNA
NCTC8325_00393	2.43	NCTC8325_00393	tRNA-Ser
NCTC8325_02602	2.43	NCTC8325_02602	exported protein
NCTC8325_01021	2.42	*hla*	alpha-hemolysin
NCTC8325_02714	2.35	*icaC*	intercellular adhesion protein icaC
NCTC8325_02556	2.31	*yvnA*	MarR family transcriptional regulator
NCTC8325_01474	2.29	*comGD*	Late competence protein ComGD%2C access of DNA to ComEA
NCTC8325_02541	2.27	*sarT*	Transcriptional regulator SarT
NCTC8325_00750	2.26	NCTC8325_00750	membrane protein
NCTC8325_00404	2.25	NCTC8325_00404	16S ribosomal RNA
NCTC8325_02720	2.22	*hisB*	imidazoleglycerol-phosphate dehydratase
NCTC8325_00174	2.21	*thlA*	3-ketoacyl-CoA thiolase
NCTC8325_01706	2.2	*sigS*	RNA polymerase sigma factor sigS
NCTC8325_02119	2.2	NCTC8325_02119	Uncharacterised protein
NCTC8325_00970	2.19	NCTC8325_00970	Uncharacterised protein
NCTC8325_01150	2.19	NCTC8325_01150	Uncharacterised protein
NCTC8325_00392	2.18	*gltD*	Glutamate synthase [NADPH] small chain
NCTC8325_01748	2.17	*splA*	serine protease SplA
NCTC8325_02462	2.08	*sbi*	IgG-binding protein SBI
NCTC8325_01016	2.07	NCTC8325_01016	membrane protein
NCTC8325_02723	2.05	*hisG*	ATP phosphoribosyltransferase subunit
NCTC8325_01866	2.01	NCTC8325_01866	phage major tail protein%2C TP901-1 family%3B phi 11 orf39
NCTC8325_00391	1.98	*gltB*_1	glutamate synthase [NADPH] large subunit
NCTC8325_00175	1.97	NCTC8325_00175	Enoyl-CoA hydratase / Enoyl-CoA hydratase [valine degradation] / 3-hydroxyacyl-CoA dehydrogenase
NCTC8325_00521	1.95	*vraX*	protein vraX
NCTC8325_00165	1.94	*uhpT*	sugar phosphate transporter
NCTC8325_02712	1.93	*icaD*	Polysaccharide intercellular adhesin (PIA) biosynthesis protein IcaD

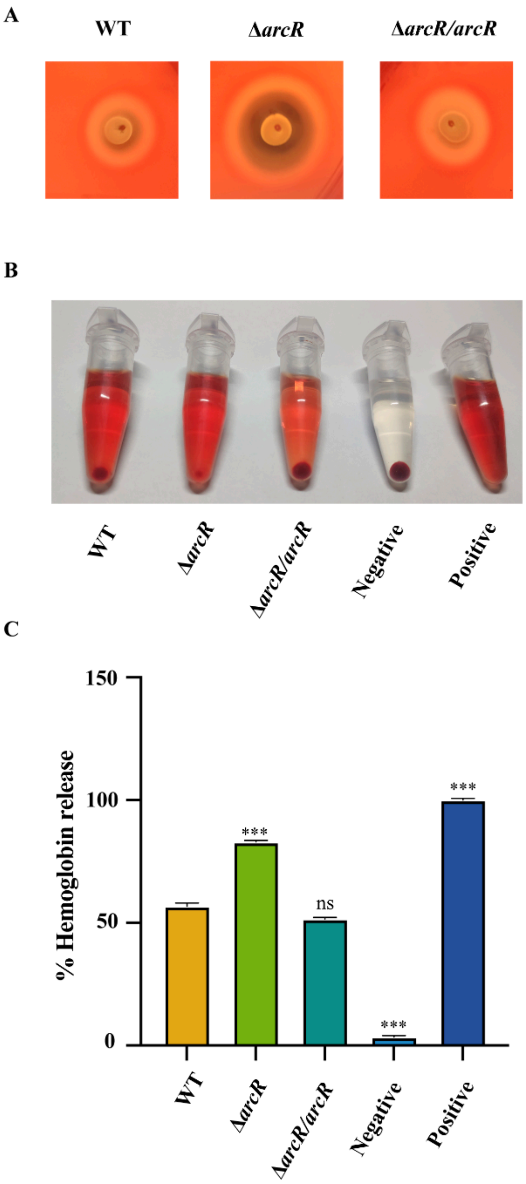

Supplementary Figure S1 ArcR regulates the hemolytic activity of *S. aureus* USA300. (A) Hemolytic activities of the WT, Δ*arcR* mutant, and complemented *arcR* mutant were evaluated on SBA plates. (B) Hemolytic activities of the WT, Δ*arcR* mutant, and complemented *arcR* mutant were determined by incubating samples with 3% sheep red blood cells, PBS and ddH_2O were used as negative control and positive control, respectively. (C) Hemolytic activities of the WT, Δ*arcR* mutant, and complemented *arcR* mutant were determined by measuring the absorption of supernatants at 543 nm. The error bars indicate the standard errors of the means of three biological replicates. ***$P < 0.001$, analyzed by one-way ANOVA.

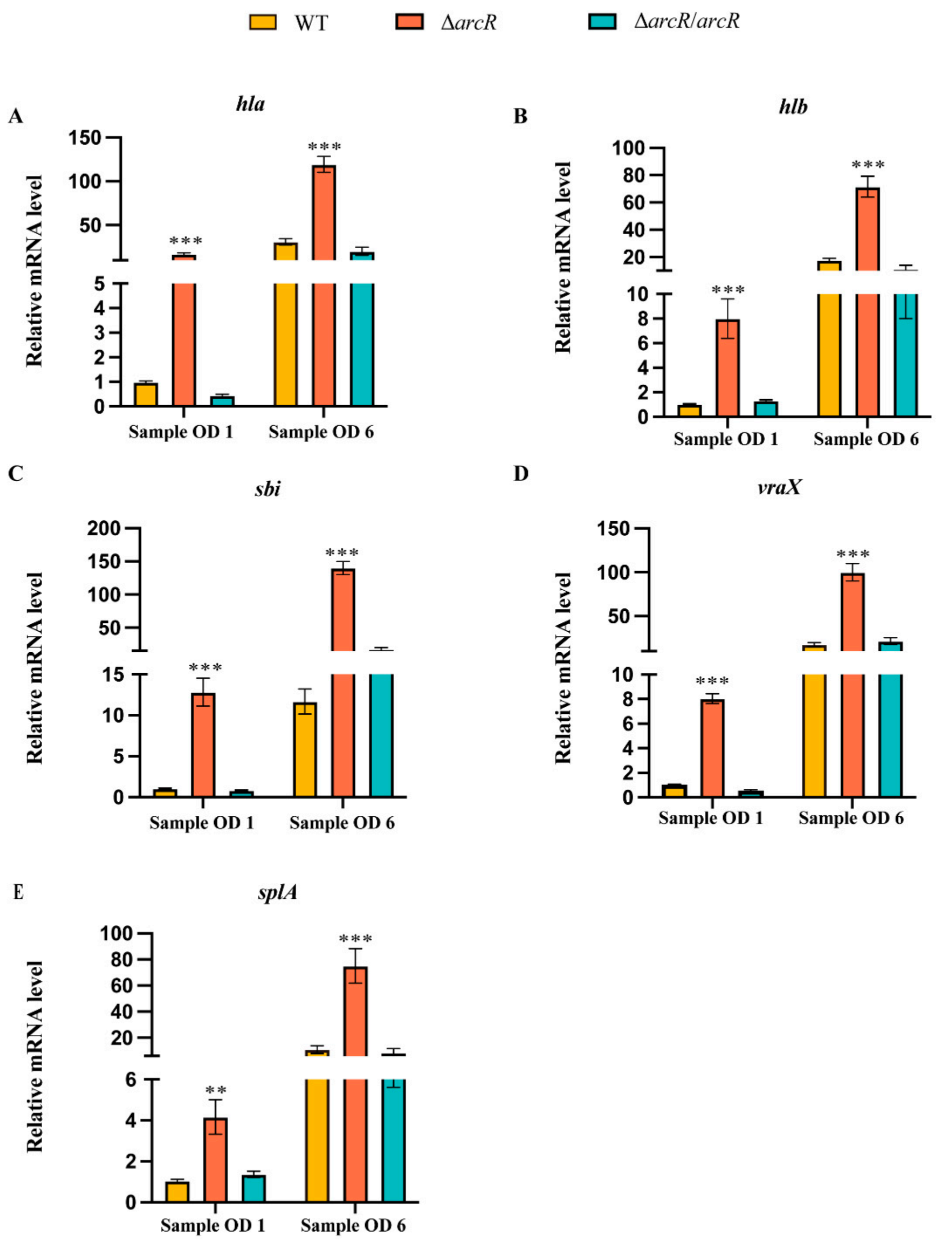

Supplementary Figure S2 ArcR negatively regulates the transcription of hemolytic genes in *S. aureus* USA300. The transcript levels of *hla* (A), *hlb* (B), *sbi* (C), *vraX* (D) and *splA* (E) in the WT of *S. aureus* USA300, Δ*arcR* mutant, and complemented *arcR* mutant at different growth phases. **, $P < 0.01$, ***$P < 0.001$, analyzed by one-way ANOVA.

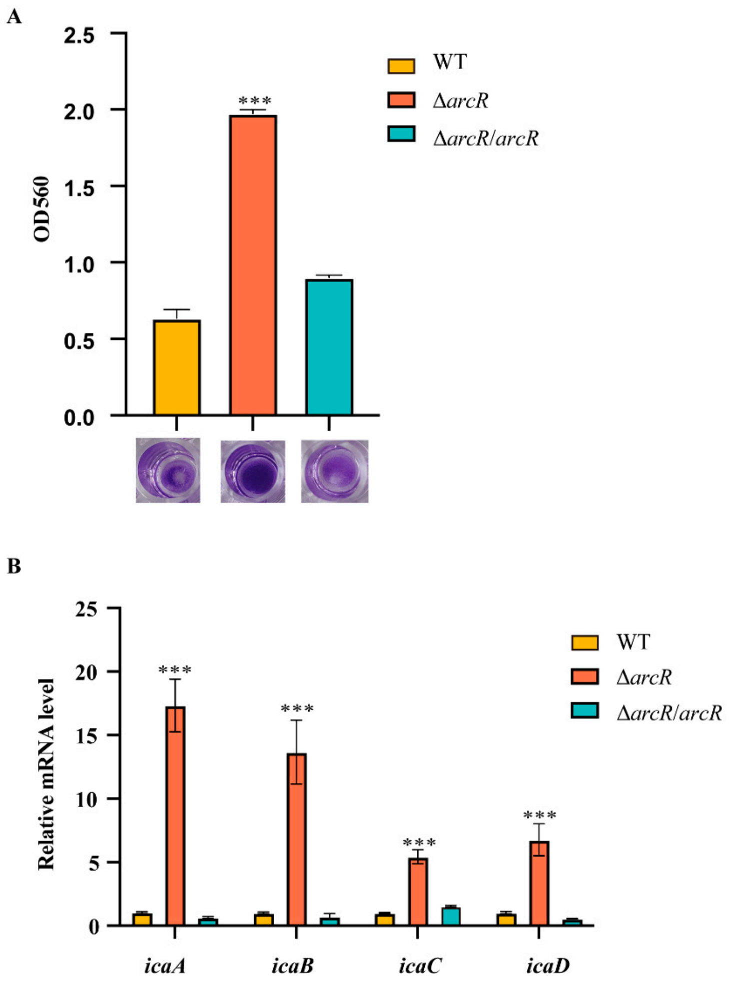

Supplementary Figure S3 Roles of ArcR in biofilm formation of *S. aureus* USA300. (A) Biofilm formation of the WT of *S. aureus* USA300, Δ*arcR* mutant, and complemented *arcR* mutant was detected by 96-well microtiter plate assays. ***P < 0.001, compared to each other by one-way ANOVA. (B) The transcript levels of *icaA*, *icaB*, *icaC* and *icaD* in the WT of *S. aureus* USA300, Δ*arcR* mutant, and complemented *arcR* mutant detected by qRT-PCR. ***P < 0.001, analyzed by one-way ANOVA.

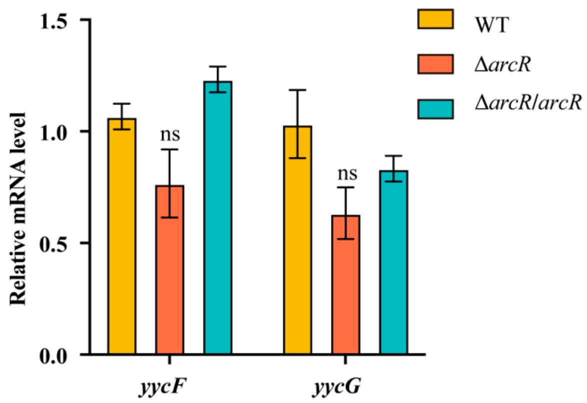

Supplementary Figure S4 The transcriptional levels of *yycFG* in *S. aureus* NCTC8325.

The transcript levels of *yycF* and *yycG* (B) in the WT, Δ*arcR* mutant, and *arcR* complementary strains. The error bars indicate the standard errors of the means of three biological replicates. ns: no significant difference. ns, indicated no statistical difference, analyzed by one-way ANOVA.

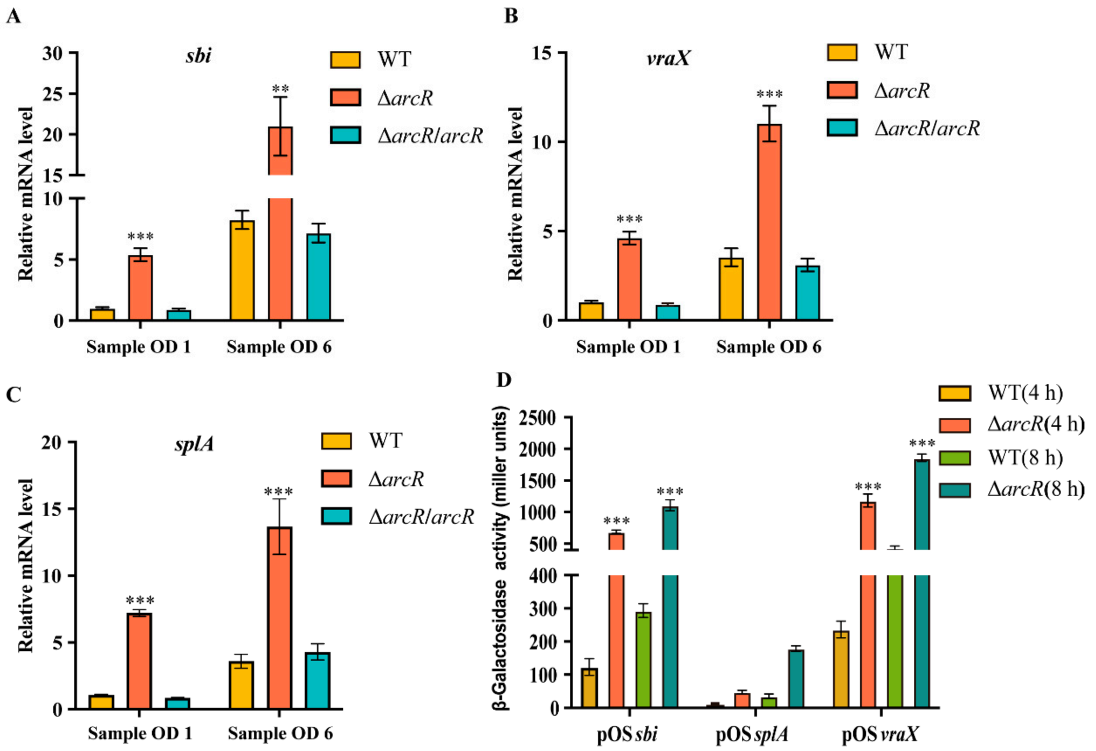

Supplementary Figure S5 ArcR negatively regulates the transcriptional of virulence genes in *S. aureus* NCTC8325.

The transcript levels of *sbi* (A), *vraX* (B) and *splA*(C)in the WT NCTC8325, Δ*arcR* mutant, and *arcR* complementary strains at different growth phases. (D) The β-galactosidase activities of *sbi*, *vraX* and *splA* promoter in the WT and Δ*arcR* mutant strains. Cells were collected at 4 and 8 h, and the β-galactosidase activity was detected with ONPG. The error bars indicate the standard errors of the means of three biological replicates. **P < 0.01, ***P < 0.001, analyzed by one-way ANOVA.
